# Supplementary material for: The interactome of intact mitochondria by cross-linking mass spectrometry provides evidence for coexisting respiratory supercomplexes
Source: Mol Cell Proteomics. 2017 Dec 8;17(2):216–32. doi: 10.1074/mcp.RA117.000470 (PMC5795388; doi:10.1074/mcp.RA117.000470)
Supplement: Supplemental Data [file supp_RA117.000470_133922_0_supp_23978_fzffwf.zip › spectra_annotation/mito_DR_spectra_annotation/327-1-10-1-14-1.pdf]

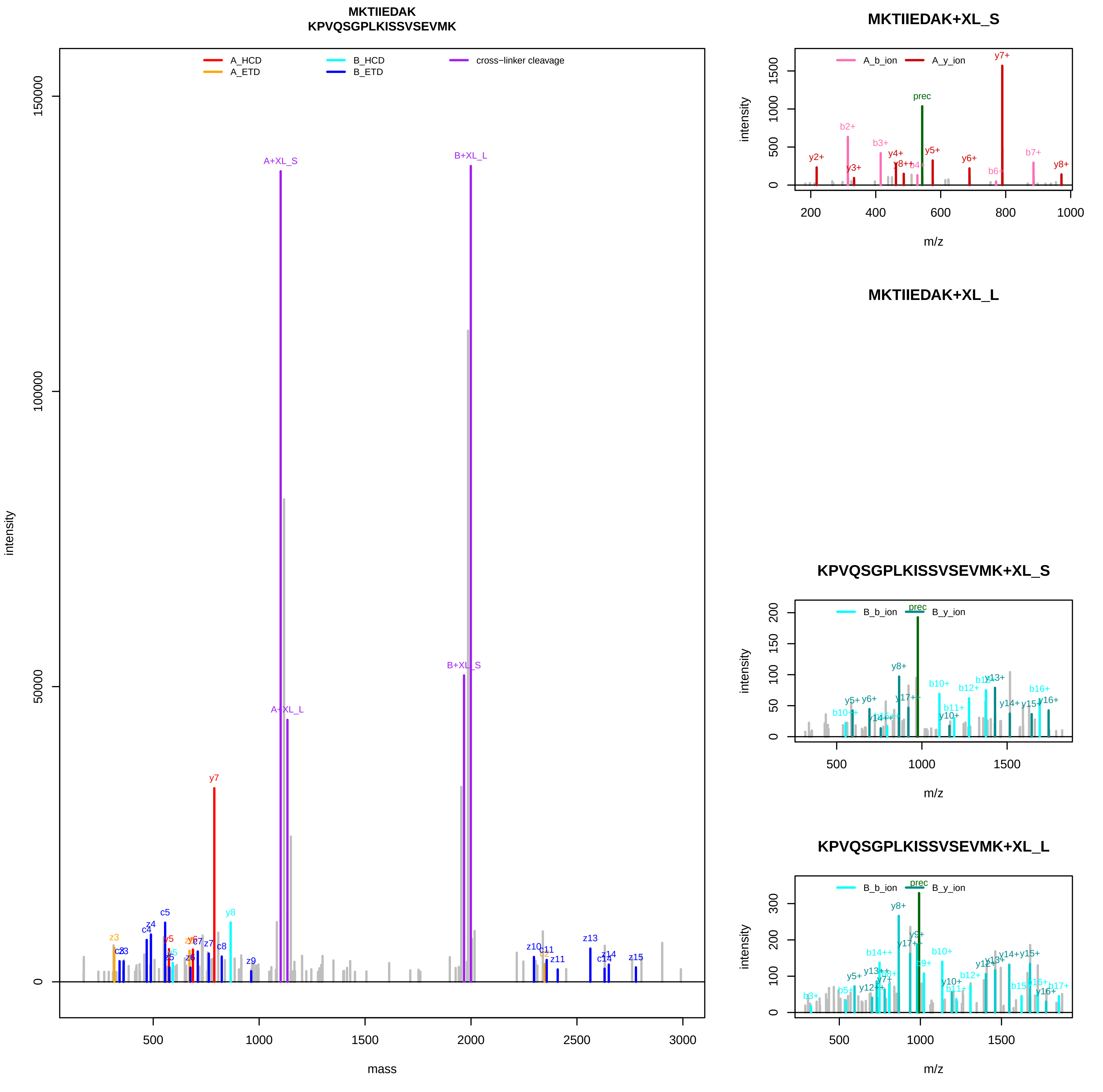

MKTIIEDAK
KPVQSGPLKISSVSEVMK
A_HCD
A_ETD
B_HCD
B_ETD
cross−linker cleavage
A+XL_S
B+XL_L
B+XL_S
A+XL_L
y7
y8
c5
z4
c4
z3
y5
z9
z10
c11
z11
z13
z14
c14
z15
intensity
mass
MKTIIEDAK+XL_S
A_b_ion
A_y_ion
prec
y7+
b2+
b3+
y2+
y3+
y4+
y5+
y6+
b6+
b7+
y8+
m/z
MKTIIEDAK+XL_L
KPVQSGPLKISSVSEVMK+XL_S
B_b_ion
B_y_ion
y8+
b10+
b12+
y13+
b16+
y5+
y6+
y17++
y14+
y15+
y16+
b11+
y10+
KPVQSGPLKISSVSEVMK+XL_L
b14++
b10+
y12+
b12+
y14+
y15+
b15+
b16+
b17+
y16+
b3+
b5+
